# Association between goiter and type 2 diabetes in a Chinese population: a cross-sectional study using CHNS data

**DOI:** 10.3389/fendo.2026.1763534

**Published:** 2026-04-07

**Authors:** Jiajia Song, Xiaofang Han, Kemei Liu, Fei Zhai, Dechao Yin, Xiaohuan Zhu, Ting Hu

**Affiliations:** Department of Endocrinology, The Second People’s Hospital of Hefei, Hefei Hospital Affiliated to Anhui Medical University, Hefei, China

**Keywords:** association, CHNS, goiter, receiver operating characteristic, T2DM

## Abstract

**Aims:**

The comorbidity of multiple diseases is receiving growing attention, and the coexistence of goiter and type 2 diabetes mellitus (T2DM) is an important one. Thus, this study aims to utilize the widely representative China Health and Nutrition Survey (CHNS) database for relevant investigations.

**Methods:**

This study adopted a pooled cross-sectional design, assessing the goiter-T2DM association using data from ten survey waves of the CHNS (1989-2018). Adjusted covariates included demographics (age, gender, education), anthropometry (BMI), lifestyle (smoking), and clinical conditions (hypertension). Univariate and multivariate logistic regression models were used to evaluate the association of goiter with T2DM. Additionally, multivariable-adjusted Receiver Operating Characteristic (ROC) curves and smooth curves (via Generalized Additive Models, GAM) were constructed to investigate the association between goiter and T2DM. The ROC analysis was employed to evaluate the combined predictive value of the multivariable model, including goiter and all adjusted covariates, for T2DM risk.

**Results:**

Among 63,848 eligible participant-wave observations, 2,109 were identified as T2DM and 155 as goiter. After adjusting for covariates, a positive association between goiter and increased risk of T2DM was observed (odds ratio [OR]= 2.62, 95% confidence interval [CI]: 1.29-4.81). The multivariable model (goiter plus covariates) yielded an AUC of 0.809, representing the collective discriminatory power rather than goiter’s independent performance. Given the cross-sectional design, these results reflect statistical associations and should be interpreted with caution regarding clinical utility.

**Conclusions:**

This study identifies a positive cross-sectional association between goiter and T2DM. Given the study’s observational nature, causal or predictive inferences are precluded, highlighting the need for longitudinal research to validate this relationship and explore potential mechanisms.

## Introduction

Goiter, characterized by the pathological enlargement of the thyroid gland, and type 2 diabetes mellitus (T2DM), a chronic metabolic disorder involving hyperglycemia and insulin resistance, both represent significant global health challenges (1, 2). Epidemiological investigations have frequently observed a high prevalence of thyroid disorders among individuals with diabetes, particularly in iodine-deficient regions (3, 4). While the interplay between these two endocrinopathies is clinically recognized, the precise nature of their co-occurrence requires further elucidation.

A critical conceptual distinction exists between functional thyroid dysfunction—characterized by abnormal thyroid hormone or TSH levels—and goiter as a morphological condition. Much of the existing literature focuses on the metabolic impacts of hormonal fluctuations or autoimmune thyroid diseases. However, goiter represents a structural abnormality that can manifest even in euthyroid individuals (5). Despite the clinical relevance of thyroid morphology, the independent association between goiter per se and T2DM risk has remained secondary to studies on functional disorders, leaving a significant gap in the understanding of the morphological-metabolic relationship. Consequently, significant research efforts are currently directed toward developing innovative therapeutic strategies and elucidating novel pathogenetic pathways. Beyond established treatments, numerous pharmacological agents are undergoing clinical investigation, including those targeting the incretin system and thyroid hormone mimetics designed to enhance metabolic efficiency and insulin sensitivity (4, 6). Specifically, research into the interplay between thyroid hormone receptors and glucose homeostasis has paved the way for novel compounds that aim to modulate metabolic rates without the adverse effects of traditional systemic hormonal therapy (7). These ongoing developments signify a shift toward more integrated and personalized metabolic management.

Currently, large-scale, population-based evidence examining thyroid enlargement as a structural condition in relation to T2DM remains limited, particularly within the Chinese population. Previous research utilizing the China Health and Nutrition Survey (CHNS) database has identified various metabolic risk factors; however, the potential for goiter to serve as an independent marker for T2DM after adjusting for comprehensive clinical covariates has not been rigorously tested. Addressing this knowledge gap is essential to determine whether thyroid morphological changes correlate with glucose metabolism dysregulation independently of traditional risk factors. Current pharmacological interventions for hyperglycemia encompass biguanides, sulfonylureas, α-glycosidase inhibitors, thiazolidinediones, and insulin. In addition to these traditional agents, newer therapeutic classes—specifically sodium-glucose cotransporter-2 (SGLT2) inhibitors, glucagon-like peptide-1 (GLP-1) receptor agonists, and dual GLP-1/glucose-dependent insulinotropic polypeptide (GIP) receptor agonists (8–10)—are now widely utilized in clinical practice for the management of type 2 diabetes (6).

The present study utilized the CHNS database covering ten survey waves from 1989 to 2018 to conduct an exploratory investigation into the association between goiter and T2DM in a Chinese cohort. By analyzing this cross-sectional data, the objective is to evaluate whether goiter is significantly linked to an increased risk of T2DM. This research aims to provide a baseline epidemiological understanding of the goiter-T2DM interplay, which may offer valuable insights for metabolic risk stratification and inform future longitudinal studies into the underlying biological mechanisms.

## Methods

### Study design and participants

This study adopted a pooled cross-sectional design, incorporating data from ten survey waves (1989–2018) of the CHNS. This approach was employed to maximize the sample size and enhance statistical power. The analytic unit was defined as the participant-wave observation; individuals participating in multiple survey rounds contributed multiple observations to the dataset. Covariates across different waves were harmonized through unified coding rules and the standardization of measurement units to ensure longitudinal comparability. Furthermore, survey wave and regional structure were incorporated into the models to account for temporal trends and geographical variations in disease detection and environmental exposures.

The final analytic sample was derived through a systematic stepwise exclusion process (**Supplementary Table 1**). Initially, 82,325 observations with non-missing outcome data were extracted. First, we excluded 7,880 records with missing or invalid goiter (exposure) data. From the remaining 74,445 records, we sequentially excluded observations with missing values for: age (n=1), race (n=2,374), smoking status (n=1,128), and hypertension (n=171). Next, we further removed records missing anthropometric data (height: n=532; weight: n=401) and socioeconomic variables (education: n=2,427; medical insurance: n=295; and employment status: n=3,268). This sequential process resulted in a final analytic sample of 63,848 participant-wave observations (total excluded n=18,477). Sampling weights were not applied, as the study prioritizes the estimation of biological associations rather than population-level prevalence. This unweighted, model-based framework provided greater statistical efficiency for controlling confounding factors within the pooled dataset. The study adheres to the ethical guidelines of the Helsinki Declaration, with approval from relevant Institutional Review Boards. All participants provided informed consent, ensuring compliance with ethical standards for research involving human subjects.

### Diagnosis of goiter and T2DM

Goiter was identified through standardized physical examinations conducted by trained physicians during the each survey visit across the study period (1989–2018). The diagnostic procedure involved systematic visual inspection and palpation of the thyroid gland, following the World Health Organization (WHO) grading criteria. Goiter was defined as a thyroid lobe larger than the terminal phalanx of the participant’s thumb (Grade 1: palpable but not visible; Grade 2: visible and palpable). Goiter morphology was further classified as diffuse or nodular. All examiners underwent rigorous, unified training prior to the survey to ensure high inter-rater reliability and consistency in assessment protocols.

The primary outcome, T2DM, was defined based on data collected during the survey visit to ensure strict temporal alignment between exposure and outcome ascertainment. This cross-sectional alignment ensures that goiter status and T2DM status were measured simultaneously for each participant. Following American Diabetes Association (ADA) guidelines, T2DM was defined by the presence of at least one of the following objective laboratory criteria: (1) fasting blood glucose (FBG) ≥ 7.0 mmol/L; or (2) glycosylated hemoglobin (HbA1c) ≥ 6.5% (48 mmol/mol). While biochemical markers (FBG and HbA1c) were primarily available in the 2009 wave, T2DM status in other waves was determined by self-reported physician diagnosis and medication use. Although the questionnaire did not explicitly distinguish between diabetes types, all participants in the analytic sample were adults (≥ 18 years); given the epidemiological context in China, these cases were classified as T2DM. Sensitivity analyses were implemented to separately evaluate biomarker-defined cases and self-reported cases to ensure the robustness of the identified associations.

### Covariates selection

The selection of covariates was guided by a conceptual framework aimed at addressing multiple pathways of metabolic confounding. Demographic characteristics, anthropometric measurements, and health conditions were included to control for established risk factors for both thyroid and metabolic diseases. Notably, physical activity and dietary factors, previously treated as exclusion criteria, were reclassified and incorporated into the multivariable models as covariates to more accurately mitigate their confounding influence. However, it is acknowledged that certain key variables, such as iodine exposure status, biochemical thyroid function (TSH and thyroid hormone levels), thyroid autoantibodies (TPOAb/TgAb), and the use of specific medications like metformin, were unavailable in the database. The absence of these factors represents a potential source of residual confounding, which is further addressed in the limitations section.

### Statistical analysis

Continuous variables were presented using mean and standard deviation [Mean (SD)], and t test was utilized for comparison between T2DM group and non-T2DM group. Categorical variables were presented as percentage with proportion [n (%)], and chi-square (χ²) test was used for the comparison. Categorical variables were incorporated into the models as dummy variables. To ensure clinical interpretability, the “healthy” or “predominant” state was generally selected as the reference category. Specifically, for hypertension, “No” was used as the reference to evaluate the risk associated with the presence of the condition. Similarly, “Han” was the reference for ethnicity, and “Employed” (Work: Yes) served as the reference for employment status. These coding strategies were applied consistently across both univariate and multivariate analyses.

Univariate and multivariate logistic regression models were employed to investigate the association between goiter and T2DM. The ROC curve was constructed based on the predicted probabilities derived from the full multivariable logistic regression model (Model 3), which incorporated goiter status alongside all adjusted covariates (including age, sex, BMI, and hypertension). This analysis was implemented using the “pROC” package in R, utilizing the roc function to calculate the Area Under the Curve (AUC) based on precise predicted probabilities. To ensure the reliability of the findings, an internal validation procedure was conducted through a 500-iteration bootstrap resampling method, and model calibration was assessed to evaluate the agreement between predicted and observed outcomes. The reported AUC represents the collective discriminatory ability of the comprehensive model rather than the independent predictive value of goiter alone. To further explore the functional relationship between continuous covariates (such as age or BMI) and the probability of T2DM, a Generalized Additive Model (GAM) with cubic splines was utilized. While goiter is treated as a binary variable, the smooth curves were generated to visualize how the overall risk profile shifts in relation to thyroid status and other continuous metabolic factors. Regarding model specification, height, weight, and BMI were all included in the fully adjusted model to account for distinct aspects of body composition and physiological load. To address concerns of redundancy and potential multi-collinearity, the Variance Inflation Factor (VIF) was calculated for all variables. All VIF values remained below 3, indicating no significant multi-collinearity that would destabilize the estimates. Furthermore, sensitivity analyses were conducted comparing the primary model with a reduced model incorporating BMI alone. The results showed no significant differences in the odds ratios (OR) or 95% confidence intervals (CI) for goiter (P > 0.05), confirming that the simultaneous inclusion of these anthropometric variables did not bias the core findings.

A model-based approach was adopted for the association analysis. Although the CHNS utilized a complex multi-stage sampling design, sampling weights were not applied in this study. This decision was based on the premise that unweighted models are often preferred in epidemiological studies focusing on association estimation rather than population prevalence, as they can provide more stable estimates with higher statistical power when adjusted for design-related covariates. All logistic regression models were fitted using the standard glm function in R (version 4.2.2). Baseline characteristics were summarized using the tableone package (version 0.13.2). The association between goiter and T2DM was visualized via forest plots using the forestplot package (version 3.1.1). Smooth curve fitting was performed using ggplot2 (version 3.4.3) with a Generalized Additive Model (GAM) framework to evaluate potential non-linear trends. Missing data were handled using a complete-case analysis approach, as the missingness proportion was below the 10% threshold for most variables. While multiple adjustment models were employed, the risk of false-positive findings due to multiple testing is acknowledged, and results were interpreted based on the consistency of the odds ratios across hierarchical models. Missing data handling was systematically addressed. A complete-case analysis approach was adopted, where participants with missing values in any of the primary variables (including BMI, height, weight, and smoking status) were excluded. This strategy was deemed appropriate as the proportion of missingness for the majority of covariates was relatively low (<10%), and the remaining analytic sample size (n=63,848) provided sufficient statistical power to detect meaningful associations. A detailed summary of missing data by variable is provided in **Table 1**. The potential impact of sparse exposure prevalence on estimate stability was addressed. Given the relatively small number of goiter cases, particularly within the T2DM group (n=11), Firth’s penalized likelihood logistic regression was employed to mitigate sparse-data bias and ensure more robust odds ratio (OR) estimates and confidence intervals. Regarding the longitudinal nature of the CHNS, all participant-wave observations across the study period (1989–2018) were treated as independent samples in the current analysis. To account for potential temporal variations and differences across survey rounds, survey wave was incorporated into the multivariate models as a categorical covariate. This approach controlled for shifts in disease patterns and detection methods over the 30-year study period. To formally evaluate the degree of within-person correlation, we calculated the Intraclass Correlation Coefficient (ICC). The resulting ICC of 0.6923 indicated that a substantial proportion of the total variance was attributable to between-participant differences, confirming the non-independence of repeated measurements. To account for this clustering within individuals, we employed cluster-robust standard errors (CRSE) in all multivariable logistic regression models. Participant ID (idind) was used as the clustering variable to adjust the standard errors, ensuring valid statistical inference despite the repeated-measure nature of the data. All adjusted models were implemented using the sandwich and lmtest packages in R.

**Table 1:** Detailed summary of missing data by variable.

Variable	Data count	Number of missing data	Missing proportion
idind	82325	0	0%
wave	82325	0	0%
height	75768	6557	8%
weight	75446	6879	8.40%
bmi	75344	6981	8.50%
high_blood_pressure	82118	207	0.30%
smoking	79742	2583	3.10%
diabetes	82325	0	0%
edu	78466	3859	4.70%
urban	81962	363	0.40%
insurance	80749	1576	1.90%
work	77061	5264	6.40%
age	82324	1	0%
race	79690	2635	3.20%
sex	82325	0	0%

Due to the low prevalence of goiter in the study population (0.24%), standard logistic regression models may be susceptible to sparse-data bias or quasi-separation, which can lead to inflated odds ratios (ORs) and unstable confidence intervals (CIs). To address this, Firth’s penalized likelihood regression was employed as a sensitivity analysis. This involved testing the association between goiter and T2DM across three hierarchical adjustment models: an unadjusted model (Model 1), a model adjusted for demographic characteristics (Model 2), and a fully adjusted model incorporating socio-economic, anthropometric, and clinical factors (Model 3). Additionally, the stability of the odds ratio (OR) was assessed to ensure that the core association remained significant across different adjustment strategies, thereby mitigating concerns regarding the impact of the restrictive exclusion criteria on the study’s generalizability.

## Results

### Characteristics of participants

**Figure 1:**
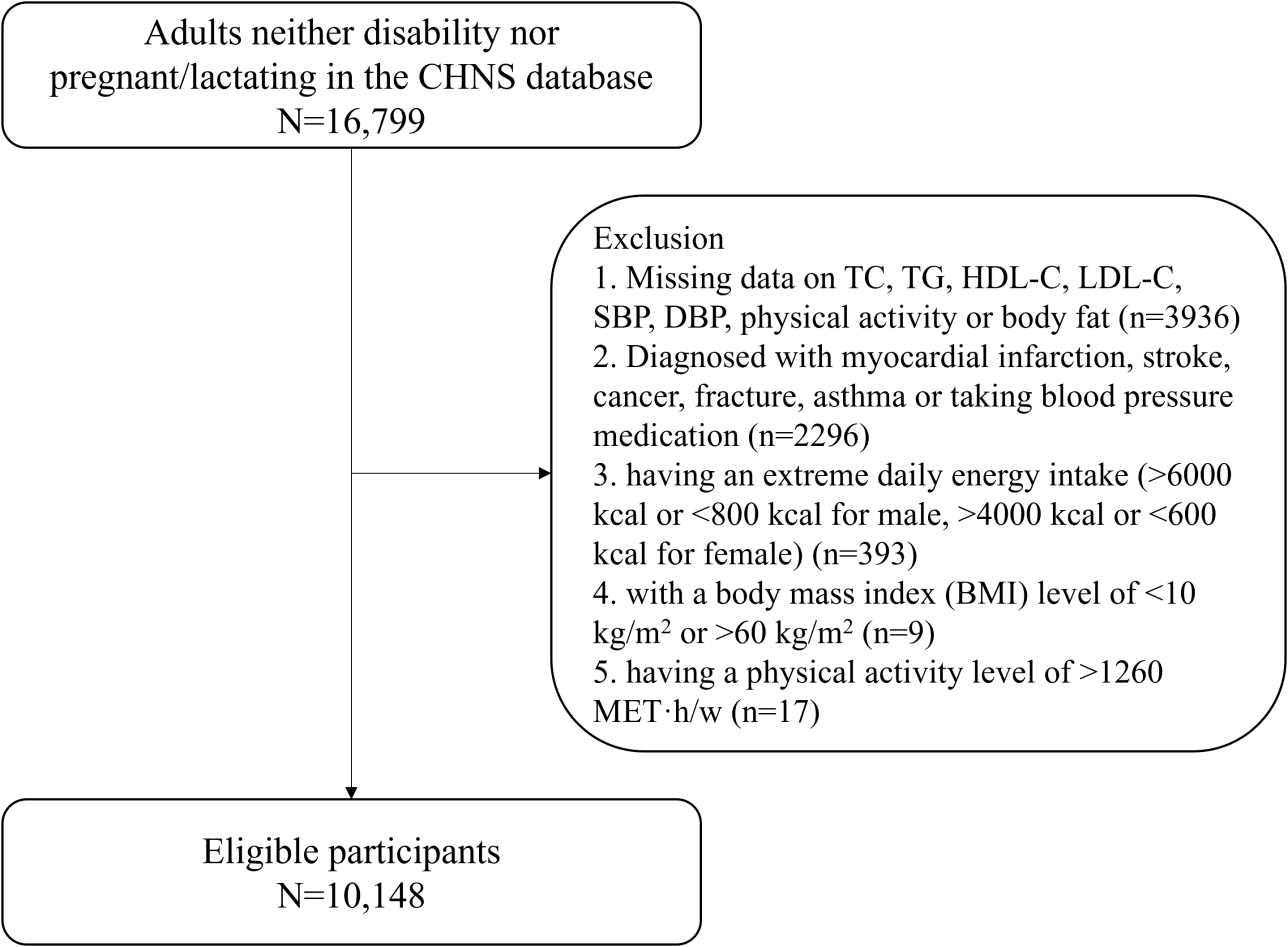
Flowchart outlining participant selection from the CHNS database: 16,799 adults screened, with exclusions for missing data, certain diagnoses, extreme energy intake, abnormal BMI, and high physical activity; 10,148 remain eligible.

**Figure 1** presents the flowchart of participant screening. From an initial pool of 82,325 observations, 63,848 met the criteria for the final analysis. The T2DM group consisted of 2,109 observations, while the non-T2DM group included 61,739 observations, as shown in **Table 2**. An assessment of data missingness revealed that proportions ranged from 0% for core variables (age, sex, and T2DM status) to a maximum of 8.5% for anthropometric measures such as BMI (**Table 1**). Missingness was primarily concentrated in height (8.0%), weight (8.4%), and employment status (6.4%). Sensitivity analyses demonstrated that the exclusion of these individuals did not significantly alter the primary association between goiter and T2DM, suggesting that the risk of substantial selection bias due to missing data is minimal.

**Table 2:** Characteristics of participants in T2DM group and non-T2DM group.

Variables	Non-T2DM (n=61739)	T2DM (n=2109)	*P*
Age, years, mean (SD)	47.33 (15.68)	59.87 (11.89)	<0.001
Ethnicity, n (%)			<0.001
Han	55822 (90.4)	1996 (94.6)	
Other	5917 (9.6)	113 (5.4)	
Gender, n (%)			0.018
Female	32430 (52.5)	1052 (49.9)	
Male	29309 (47.5)	1057 (50.1)	
Educational level, n (%)			0.012
Middle school or vocational	32951 (53.4)	1059 (50.2)	
Primary school or below	24209 (39.2)	873 (41.4)	
University or higher	4579 (7.4)	177 (8.4)	
Place of residence, n (%)			<0.001
Rural	39281 (63.6)	984 (46.7)	
Urban	22458 (36.4)	1125 (53.3)	
Health insurance, n (%)			<0.001
Yes	37677 (61.0)	1760 (83.5)	
No	24062 (39.0)	349 (16.5)	
Employment status, n (%)			<0.001
Yes	37871 (61.3)	678 (32.1)	
No	23868 (38.7)	1431 (67.9)	
Height, cm, mean (SD)	160.95 (8.17)	161.17 (8.16)	0.222
Weight, kg, mean (SD)	60.04 (10.19)	65.05 (9.75)	<0.001
BMI, kg/m^2^, mean (SD)	23.12 (3.23)	25.02 (3.29)	<0.001
Hypertension, n (%)			<0.001
Yes	6284 (10.2)	878 (41.6)	
No	55455 (89.8)	1231 (58.4)	
Smoking, n (%)			<0.001
Current	16892 (27.4)	489 (23.2)	
Never	43106 (69.8)	1483 (70.3)	
Past	1741 (2.8)	137 (6.5)	
Goiter, n (%)			0.015
Yes	144 (0.2)	11 (0.5)	
No	61595 (99.8)	2098 (99.5)	

Statistics included t test and chi-square test.

T2DM, type 2 diabetes mellitus, SD, standard deviation, BMI, body mass index.

Comparison of characteristics in participants between T2DM group and non-T2DM group was shown in **Table 2**. Among the total population, 2,109 had T2DM and 155 had goiter. The average age of patients with T2DM was significantly higher than those without T2DM (59.87 years vs. 47.33 years). The proportion of people with the ethnicity of Han was significantly higher than that in non-T2DM group (94.6% vs. 90.4%). Standardized difference analysis revealed that the most pronounced disparity between groups was in age (SMD = 0.901), representing a large effect size (**Table 3**). Moderate-to-large effects were also observed for hypertension prevalence (SMD = 0.769), employment status (SMD = 0.612), BMI (SMD = 0.585), and health insurance coverage (SMD = 0.517). In contrast, differences in sex (SMD = 0.053), ethnicity (SMD = 0.161), and goiter prevalence (SMD = 0.047) were negligible (SMD < 0.2). Regarding data integrity, missing proportions for key covariates were assessed prior to exclusion. Missingness was highest for BMI (8.5%) and height (8.0%), while other variables, such as smoking status (3.1%) and education level (4.7%), showed lower proportions. Complete-case analysis was deemed appropriate given these distributions and the overall large sample size. More than half of individuals in non-T2DM group were female, while the proportion of male patients was higher in T2DM group (P = 0.018). The average weight and BMI levels were both significantly higher in T2DM group than those in non-T2DM group (65.05 kg vs. 60.04 kg). Patients with hypertension occupied 41.6% in T2DM group, while the number in non-T2DM group was 10.2% (P<0.001).

**Table 3:** Standardized mean differences (SMD) of participant characteristics between T2DM and non-T2DM groups.

Variable	Type	Std_Difference
height	Continuous	0.027
weight	Continuous	0.503
bmi	Continuous	0.585
high_blood_pressure: Yes	Categorical	0.769
high_blood_pressure: No	Categorical	-0.769
smoking: current	Categorical	-0.096
smoking: never	Categorical	0.011
smoking: past	Categorical	0.175
edu: Middle_or_vocational	Categorical	-0.063
edu: Primary_or_below	Categorical	0.044
edu: University_or_higher	Categorical	0.036
urban: Rural	Categorical	-0.346
urban: Urban	Categorical	0.346
insurance: No	Categorical	-0.517
insurance: Yes	Categorical	0.517
work: Yes	Categorical	-0.612
work: No	Categorical	0.612
age	Continuous	0.901
ethnicity: Han	Categorical	0.161
ethnicity: Other	Categorical	-0.161
sex: female	Categorical	-0.053
sex: male	Categorical	0.053
Goiter: No	Categorical	-0.047
Goiter: Yes	Categorical	0.047

### Association between goiter and T2DM

The associations between goiter and T2DM remained robust across sequential levels of covariate adjustment (**Table 4**). In the unadjusted model (Model 1), goiter was associated with increased odds of T2DM (OR = 2.24, 95% CI: 1.01–4.98, p=0.047). After fully adjusting for demographics, anthropometrics, clinical factors, and accounting for within-person correlation via cluster-robust standard errors, the association remained significant (Model 3: OR = 2.62, 95% CI: 1.05–6.55, p=0.040).

**Table 4:** Association between goiter and T2DM.

Exposure	model1_OR(95%_CI)	model2_OR(95%_CI)	model3_OR(95%_CI)
Goiter	2.24(95% CI: 1.01–4.98)	2.44(95% CI: 1.05–5.66)	2.62(95% CI: 1.05–6.55)
p_value	0.047	0.038	0.040

T2DM, type 2 diabetes mellitus; OR, odds ratio; CI, confidence interval.

Model 1, unadjusted model.

Model 2, adjusted for age, gender and ethnicity.

Model 3, adjusted for age, gender, ethnicity, educational level, health insurance, employment status, place of residence, height, weight, BMI, smoking status, hypertension, and survey wave.

**Figure 2:**
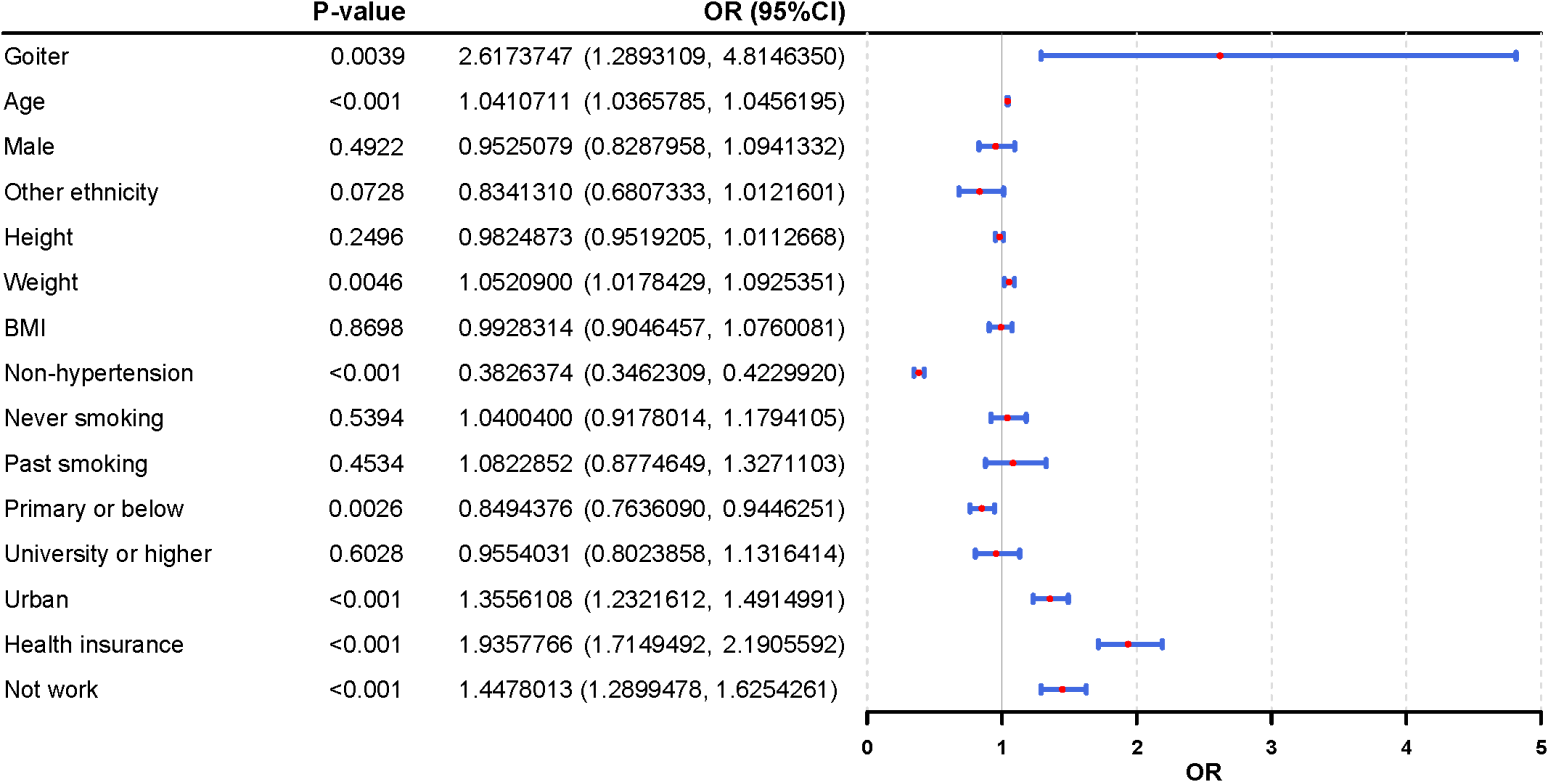
Forest plot graphic illustrating odds ratios (OR) with ninety-five percent confidence intervals for various demographic and health factors. Statistically significant variables include goiter, age, weight, non-hypertension, education, urban status, health insurance, and employment status.

These multivariable-adjusted associations were visualized in a forest plot (**Figure 2**). Beyond goiter, hypertension, advancing age, and urban residence were identified as significant risk factors for T2DM, while higher educational attainment and rural residence were associated with a lower risk.

To contextualize the clinical magnitude, absolute risk estimates were derived from the fully adjusted model. The predicted probability of T2DM was 7.097% for individuals with goiter compared to 3.294% for those without, corresponding to an absolute risk difference (ARD) of 3.803%. These data indicate that while goiter significantly elevates the relative odds of T2DM, the absolute increase in risk remains modest within this population.

Finally, sensitivity analysis using Firth’s penalized likelihood regression confirmed the stability of these findings. Even when accounting for potential sparse-data bias, goiter correlated with a significant increase in the odds of T2DM across all models (Model 3: OR = 2.715, 95% CI: 1.356–4.947, p=0.006), further supporting the robustness of the primary analysis.

### ROC and nonlinear correlation analysis

**Figure 3:**
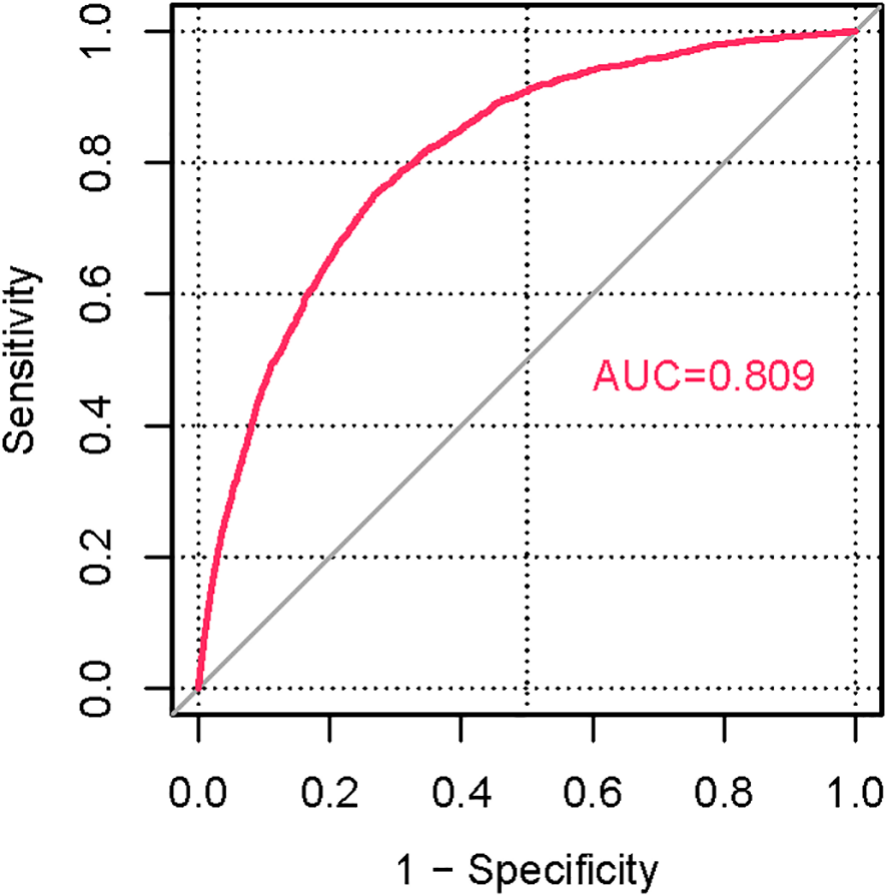
Receiver operating characteristic curve displaying sensitivity versus one minus specificity for a diagnostic test, with a red curve and area under the curve value of zero point eight zero nine indicated in red text.

**Figure 4:**
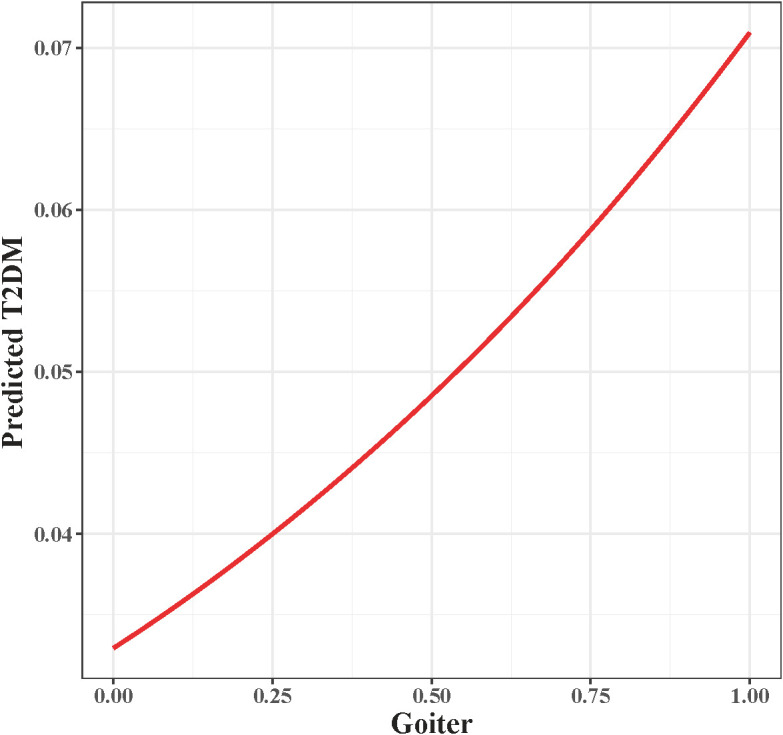
Line graph illustrating the relationship between goiter (x-axis, ranging from zero to one) and predicted type 2 diabetes mellitus (T2DM) risk (y-axis, ranging from approximately zero point zero three to zero point zero seven), with risk increasing non-linearly as goiter increases, depicted by a red curve.

To evaluate the discriminatory power of the identified associations, an ROC curve was constructed using the predicted probabilities derived from the full multivariable model. This model incorporated goiter status alongside other key covariates, including age, gender, ethnicity, BMI, smoking status, and clinical conditions. The resulting AUC of 0.809 (**Figure 3**) reflects the collective discriminatory capacity of these combined factors in predicting T2DM risk, rather than the independent predictive performance of goiter alone. Given the low prevalence of goiter in the analytic sample, this high AUC value highlights the overall efficacy of the multivariable framework, within which goiter serves as one of several significant contributors to model performance. A smooth curve was constructed to visualize the association between goiter and T2DM risk (**Figure 4**). The visualization, based on model-predicted probabilities, demonstrated a consistent elevation in the likelihood of T2DM among individuals with goiter compared to those without.

## Discussion

The medical community has increasingly recognized the importance of disease comorbidities (11). Existing studies indicate that thyroid dysfunction may contribute to DM pathogenesis by impairing both insulin secretion and sensitivity (12, 13). Epidemiological data consistently show elevated T2DM risk among patients with thyroid disorders, pointing to potential underlying physiological connections (14).The current study, utilizing the CHNS database, identified a significant positive association between goiter and T2DM. Specifically, goiter was linked to an increased risk of T2DM, with an adjusted odds ratio (OR) of 2.62 (95% CI: 1.05–6.55). This finding underscores a potential interplay between thyroid morphological changes and glucose metabolism dysregulation, highlighting the importance of considering thyroid status within the framework of metabolic risk assessment. While thyroid dysfunction, including hypothyroidism and hyperthyroidism, has been implicated in the pathogenesis of T2DM (15–17), our analysis specifically examines the morphological aspect of goiter. It is important to distinguish between the morphological characteristics of goiter and the functional aspects of thyroid disorders, as the latter may involve different underlying mechanisms.

The positive association between goiter and T2DM identified in this study provides preliminary epidemiological evidence of a potential link between thyroid morphology and metabolic risk. However, these findings should not be interpreted as a basis for clinical screening or targeted prevention at this stage. Given the study’s cross-sectional nature, it is premature to recommend routine thyroid evaluation as a standard component of T2DM risk assessment. Instead, these results should be viewed as a signal for future research to explore whether thyroid morphological markers could eventually contribute to more refined metabolic risk stratification models.

Participants were stratified into T2DM group and non-T2DM group, as well as various covariates were examined, thus, it was better to understand the patterns of T2DM prevalence in China. In terms of gender, while no significant association of gender with T2DM was observed in this study, gender itself has been reported to influence the risk of T2DM complications through various mechanisms (18). For example, women have a relatively higher risk of CVDs and kidney diseases, whereas men are more prone to diabetic retinopathy and painless diabetic neuropathy (19, 20).Besides, underweight is more common in iodine-sufficient urban areas, while obesity is more prevalent in iodine-deficient suburban regions (21, 22). After adjusting for significant risk factors such as DM, hypothyroidism/hyperthyroidism, phosphorus intake, dairy and seafood consumption, adult women with low dietary iodine intake were associated with a higher risk of T2DM (23). Higher placental iodine concentrations are associated with a lower incidence of gestational diabetes mellitus (GDM) (24). It has been confirmed that lower placental iodine load is linked to changes in plasma insulin levels, homeostasis model assessment of insulin resistance (HOMA-IR) index, and β-cell activity (25). It is evident that various factors related to residential areas, such as sports facilities, green space coverage, and iodine levels, influence people’s health to varying degrees, particularly the risk of developing DM (26, 27). The independence of the association between goiter and T2DM, maintained after adjusting for lifestyle and socioeconomic covariates (including smoking status, physical activity, and health insurance), suggests that thyroid morphological enlargement contributes to metabolic risk through pathways not fully explained by traditional epidemiological factors. While residential environment and health insurance status are known determinants of healthcare access and diabetes prevalence, their roles in the current analysis were primarily as confounding variables. The persistent 2.62-fold risk increase indicates that the structural state of the thyroid gland itself—potentially reflecting chronic subclinical iodine insufficiency or low-grade inflammation (28)—is a critical focal point. Consequently, the interpretation should prioritize the biological interplay between thyroid morphology and insulin sensitivity, rather than peripheral factors that, while relevant to general diabetes epidemiology, do not directly clarify the goiter-T2DM link.

Rather than expanding into general lifestyle determinants, the discussion of biological mechanisms must focus on why thyroid enlargement correlates with glucose dysregulation. A plausible explanation lies in the compensatory response of the thyroid gland to systemic metabolic stress (29). The structural changes observed in goiter may coincide with altered secretion of thyroid-derived factors that influence peripheral insulin action (30). By centering the analysis on this morphological-metabolic axis, the study provides a more targeted contribution to the understanding of endocrine comorbidities, avoiding the dilution of findings with tangential epidemiological associations.

Interpretation of the findings must balance relative and absolute measures. Although the identified OR of 2.62 suggests a substantial relative increase in risk, the absolute risk difference of 3.803% provides a more grounded perspective on the clinical impact. In populations with a low prevalence of goiter, relative measures like OR can sometimes overstate the practical importance of an association. By incorporating absolute risks, the results clarify that while thyroid enlargement is a statistically significant correlate, its contribution to the overall burden of T2DM at the population level is constrained by its rarity. Although the multivariable-adjusted ROC analysis yielded an AUC of 0.809, this result reflects the collective discriminatory power of a comprehensive model including multiple covariates (age, BMI, and hypertension) rather than the independent predictive utility of goiter alone. The clinical significance of goiter as a lone predictor remains limited, and the reported AUC should be interpreted as a reflection of the model’s overall statistical fit within this specific cross-sectional cohort.The identified association between goiter and T2DM is consistent with the hypothesis that structural changes in the thyroid gland may reflect underlying metabolic disturbances. Unlike functional thyroid disorders, goiter as a morphological condition may independently correlate with insulin resistance (IR) (31, 32). Potential mechanisms include the reduction of glucose transporter 4 (GLUT4) expression in skeletal muscle and adipose tissue, a pathway often observed in states of thyroid hormone deficiency which frequently accompanies goiter formation (7, 33).

Rather than reiterating general metabolic knowledge, the specific contribution of this study lies in highlighting goiter as an independent morphological risk marker. This association may be biologically plausible through the modulation of glucose transporter 4 (GLUT4) expression. Evidence suggests that even in the absence of overt thyroid dysfunction, structural changes in the thyroid gland can coincide with reduced insulin-mediated glucose uptake, potentially through shared autoimmune or inflammatory mechanisms that target both thyroid and pancreatic tissues simultaneously (34, 35).

Autoimmune reactions targeting both pancreatic β-cells and thyroid tissue can lead to impaired insulin secretion and thyroid enlargement simultaneously (36). However, as biochemical markers of autoimmunity were not assessed in this study, these mechanisms remain hypothetical and require validation through prospective molecular investigations.

Although thyroid hormone deficiency is known to reduce GLUT4 expression and exacerbate insulin resistance, it remains speculative to attribute the current findings purely to hormonal fluctuations. Instead, the morphological changes associated with goiter may independently contribute to metabolic dysregulation. For instance, thyroid enlargement might reflect a state of chronic iodine deficiency or subclinical autoimmune activity, both of which have been hypothesized to influence systemic glucose homeostasis. Future research incorporating both thyroid ultrasound and biochemical markers is necessary to clarify whether these morphological changes act through functional pathways or through independent metabolic signaling.

In the context of clinical management, it is noteworthy that the therapeutic landscape for T2DM has evolved beyond traditional regimens. While conventional therapies are often limited by adverse effects such as hypoglycemia and weight gain (37), newer pharmacological agents, specifically sodium-glucose cotransporter-2 (SGLT2) inhibitors and glucagon-like peptide-1 (GLP-1) receptor agonists, offer superior safety and efficacy profiles. These agents are not associated with an increased risk of hypoglycemia and have demonstrated significant potential for weight reduction. Furthermore, large-scale clinical trials have confirmed that SGLT2 inhibitors and GLP-1 receptor agonists provide substantial cardiovascular protection, including significant reductions in major adverse cardiovascular events (MACE) and hospitalizations for heart failure (38). Given that patients with both goiter and T2DM may face an elevated risk of cardiovascular complications, the integration of these modern therapies into personalized treatment plans could potentially improve long-term clinical outcomes.

Furthermore, the historical concern regarding therapeutic resistance in long-term diabetes management is largely mitigated by the distinct mechanisms of action of modern therapies. SGLT2 inhibitors and GLP-1 receptor agonists offer sustained efficacy and avoid the progressive β-cell exhaustion often linked to traditional secretagogues (39). The ability of these newer agents to provide continuous cardiovascular and renal protection, independent of their glycemic effects, ensures a more stable and comprehensive therapeutic profile, effectively addressing the debate over long-term clinical durability.

While GLP-1 receptor agonists provide significant metabolic and cardiovascular advantages, their clinical application necessitates careful consideration of potential thyroid-related safety concerns. A prominent area of ongoing controversy involves the hypothesized association between GLP-1 receptor agonist therapy and an increased risk of malignant thyroid nodules, specifically medullary thyroid carcinoma (MTC) (40). Current clinical guidelines explicitly state that these agents are contraindicated in patients with a personal or family history of MTC or in those with Multiple Endocrine Neoplasia syndrome type 2 (MEN 2). Although the causal relationship remains a topic of active debate in the literature, it is imperative for clinicians to maintain vigilance regarding thyroid health when prescribing these medications, particularly in individuals presenting with pre-existing morphological abnormalities such as goiter. Given the positive association between goiter and T2DM identified in the present study, future longitudinal research is warranted to clarify whether the use of GLP-1 receptor agonists interacts with thyroid structural changes to influence long-term malignancy risk.

Findings in the current study, that is, the positive association between goiter and increased T2DM risk, may provide valuable insights for early clinical screening and intervention and implementing preventive measures in T2DM. Thus, routine follow-up for T2DM patients should incorporate thyroid function evaluation, particularly for those presenting with signs of thyroid enlargement, which may achieve more comprehensive health management and better disease control. Furthermore, integrating multiple covariates reinforced the role of goiter as an independent risk factor for T2DM. In fact, lifestyle and genetic factors may interact complexly with goiter and DM, whether incorporating additional biomarkers may further improve predictive accuracy of goiter on T2DM risk, multicenter validation studies would be necessary. Limitations could not be ignored in the results explanation.Several methodological constraints must be explicitly recognized to provide a balanced interpretation of the findings. First, the low prevalence of goiter (0.24% overall) resulted in a sparse-data scenario, with only 11 exposed cases identified within the T2DM group. While Firth’s penalized regression was utilized to stabilize the estimates and address potential quasi-separation, the small number of exposed individuals inevitably limits the precision of the odds ratios. Second, goiter was assessed solely via physical examination rather than ultrasound confirmation. This approach carries an inherent risk of exposure misclassification, as palpation may fail to detect smaller nodules or yield false positives, potentially leading to an attenuation of the observed association. Third, the lack of biochemical data on iodine status, thyroid-stimulating hormone (TSH) levels, and thyroid autoantibodies represents a significant source of residual confounding. Specifically, the inability to adjust for metformin use—a medication known to influence thyroid volume—may further impact the accuracy of the goiter-T2DM relationship. Furthermore, the inconsistency of diabetes definitions across CHNS waves (transitioning from purely self-reported data to objective biomarkers in 2009) may introduce outcome heterogeneity. Although the total sample size was standardized to 63,848 participant-wave observations to maintain internal consistency, the reliance on a pooled cross-sectional structure means that individual-level longitudinal shifts in thyroid morphology were not fully captured. Finally, the non-application of sampling weights may influence the representativeness of these findings on a national scale. Consequently, these results should be regarded as exploratory epidemiological evidence that requires validation in larger prospective cohorts incorporating standardized thyroid imaging and comprehensive biochemical markers. Due to the cross-sectional study design, sample size constraints and potential selection bias are inescapable, as well as causal association between goiter and T2DM could not be concluded. While the CHNS provides representative data, generalizability may be limited by participant selection criteria, and mechanistic experiments are needed to enhance external validity. Future multi-center studies with larger samples should validate these associations and explore underlying biology.

## Conclusion

This analysis identifies a significant statistical association between goiter and T2DM within the CHNS cohort. However, given the cross-sectional nature of the study and the low prevalence of goiter, these findings should be interpreted with caution and regarded as exploratory. To validate this observed association and fully elucidate the underlying biological mechanisms, future research must move beyond broad calls for further study and specifically address several methodological requirements. Future investigations should prioritize longitudinal designs to establish temporal relationships, utilize standardized thyroid assessments (such as ultrasound confirmation), and apply consistent diabetes definitions across different survey waves. Furthermore, multi-center studies with larger numbers of goiter cases are necessary to enhance statistical power and ensure the stability of risk estimates. Such rigorous evidence will be essential for determining whether these morphological findings can be translated into improved patient management or prevention strategies.

## Data Availability

The original contributions presented in the study are included in the article/**Supplementary Material**. Further inquiries can be directed to the corresponding author.

## Supplementary material

Supplementary Table 1 Sequential flow of sample selection. Exclusions were performed sequentially; therefore, counts in this table reflect additional records removed at each step, accounting for overlap with previously excluded categories.
