# Supplementary figures and images for: Association between goiter and type 2 diabetes in a Chinese population: a cross-sectional study using CHNS data
Source: Front Endocrinol (Lausanne). 2026 Apr 7;17:1763534. doi: 10.3389/fendo.2026.1763534 (PMC13095509; doi:10.3389/fendo.2026.1763534)

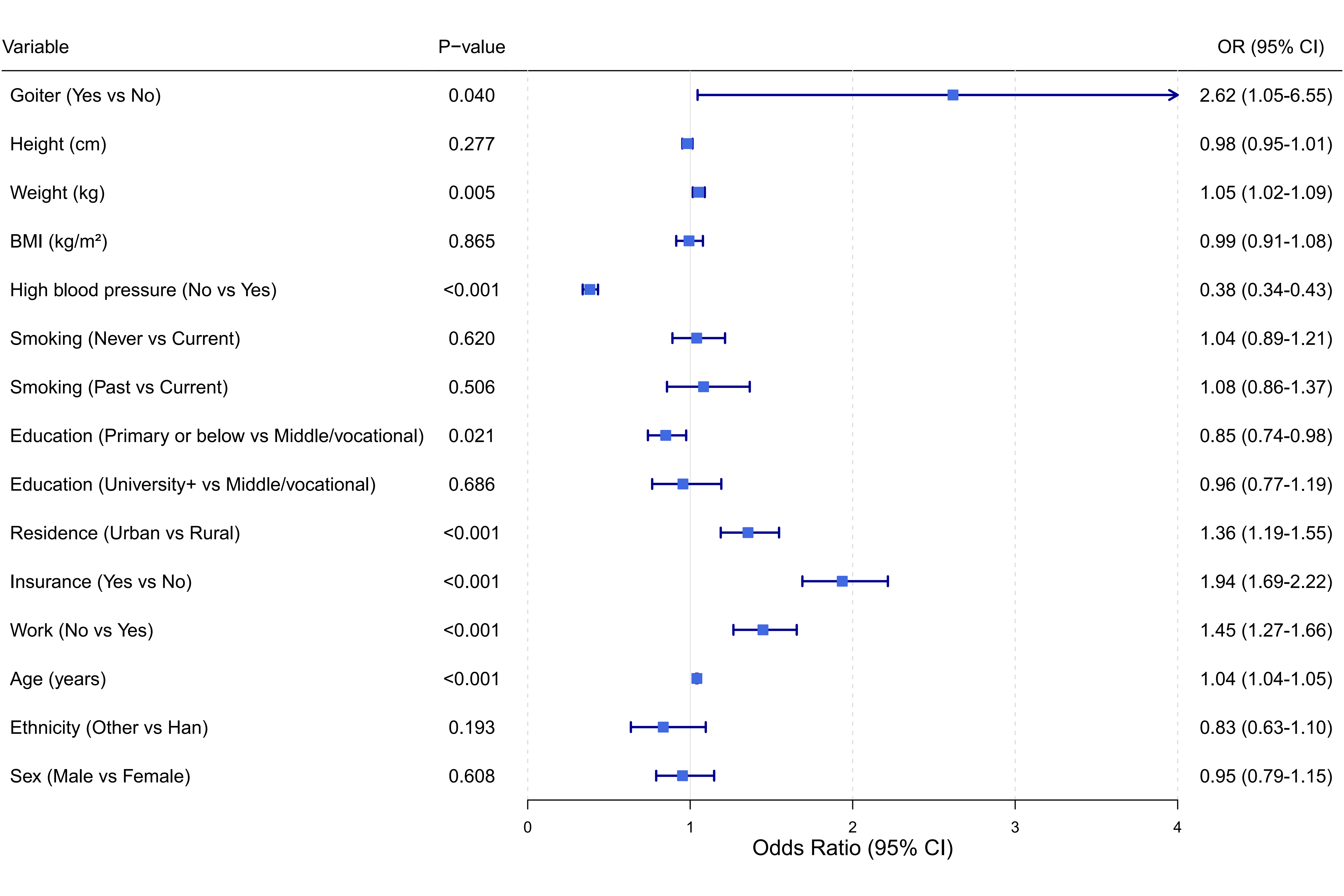

Supplement: Supplementary Table 1 — Sequential flow of sample selection. Exclusions were performed sequentially; therefore, counts in this table reflect additional records removed at each step, accounting for overlap with previously excluded categories. [file Image1.png]
